# Appendicite aigue à manifestation clinique gauche sur mésentère commun complet: à propos d’un cas

**Published:** 2010-11-18

**Authors:** Aziz Elmadil, Hanan Bouamama, Mohammed Ramil, Khalid Khattal, Abderrahman Afifi, Youssef Bouabdallah

**Affiliations:** 1 Service de chirurgie pédiatrique, CHU Hassan II de Fès, Fès, Maroc

**Keywords:** Malrotation intestinale, appendicite, fosse iliaque gauche

## Abstract

**Abstract:**

La malrotaion intestinale chez l’enfant est une anomalie rare due à un arrêt des phénomènes de rotation et d’accolement de l’intestin primitif. Elle peut être grave en cas de mésentère commun incomplet en se compliquant d’un volvulus du grêle ou asymptomatique si la rotation s’arrête à 90° se déclarant ainsi par des formes atypiques d’appendicite comme c’est le cas de notre observation. Il s’agit d’un garçon de 15 ans qui a présenté une semaine avant son admission des douleurs abdominales diffuses avec vomissements alimentaires évoluant dans un contexte fébrile et chez qui l’examen clinique avait montré une sensibilité de la fosse iliaque gauche. Le bilan biologique avait objectivé un syndrome inflammatoire biologique. Les examens radiologiques étaient en faveur d’un mésentère commun complet avec multiples adénopathies mésentériques et infiltration de la graisse mésentérique. L’exploration cœlioscopique a confirmé le diagnostic de malrotation avec appendicite aigue. L’évolution après l’appendicectomie était favorable. Si l’appendicite aigue dans sa forme habituelle est une urgence chirurgicale fréquente chez l’enfant, ses formes atypiques dans le cadre d’une malrotation intestinale ou d’un situs inversus restent très difficiles à diagnostiquer. L’imagerie moderne notamment la tomodensitométrie (TDM) abdominale puis la cœlio-chirurgie ont beaucoup contribué au diagnostic de ces forms.

## Introduction

L’appendicite aiguë est l’urgence chirurgicale la plus fréquente d’une douleur de la fosse iliaque droite chez l’enfant, son siège à gauche suite à une malrotation intestinale est extrêmement rare et donc souvent responsable d’un retard diagnostic. L’imagerie permet un diagnostic anatomique et lésionnel précis orientant donc la voie d’abord chirurgicale.

## Patient and observation

A 38 years-old woman presented in June 2009 with a one year history of progressive widespread symmetrical cutaneous thickening of the skin of the proximal upper extremities, trunk and face, arthralgias, dyspnea on exertion and 10 kg weight loss over the previous 12 months.

She had been diagnosed with type I adult-onset pityriasis rubra pilaris (PRP) at age 36 years, and had been treated with topical corticosteroids, emollients and cetirizine dichlorhydrate. Family history was negative for skin diseases. Raynaud’s phenomenon was denied.

**Figure 1:**
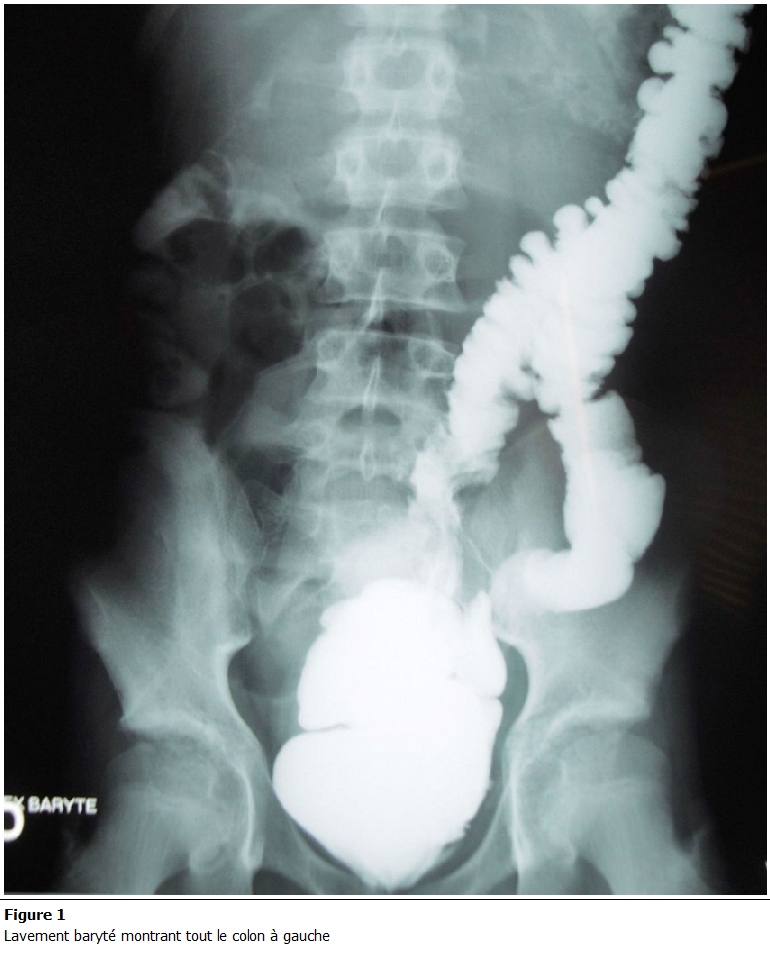
Lavement baryté montrant tout le colon à gauche

**Figure 2:**
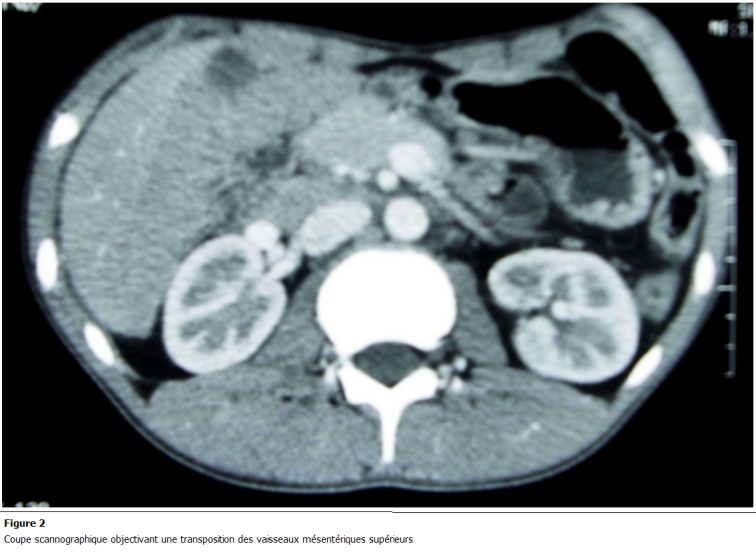
Image peropératoire révélant des adhérences entre la paroi abdominale antérieure et le carrefour iléo-caecal placé au niveau de la fosse iliaque gauche

Physical examination revealed a diffuse erythematous desquamative cutaneous eruption with diffuse skin thickening, telangiectasias ans sclerodactyly with finger flexion contractures and digital tuft loss (Figure 1). The palmoplantar surfaces were hyperkeratotic and fissured with areas of peeling (Figure 2). Mouth excursion was limited. Capillaroscopy showed avascular areas and capillary dilatations.

Laboratory tests showed the following results: the erythrocyte sedimentation rate (ESR) 12 mm/h, normal haemoglobin, white blood cells count (WBC) and platelet count., SGOT 32 IU/l, SGPT 44 IU/l. Renal function was normal. The serum calcium, phosphate, protein and creatine kinase level were within the normal limits. Antinuclear antibodies (ANA) were present at 1/1280, in a nucleolar pattern; anti PM-Scl positive.

Histological evaluation of a lesional skin biopsy revealed orthokeratosis and confluent granular layer in the epidermis, and a perivascular lymphohistiocytic cell infiltrate in the papillary dermis consistent with PRP.

Chest radiograph, echocardiogram and electrogram were within normal limits but pulmonary function revealed moderate restrictive disease. Hand radiographs revealed resorption of the distal tufts of several fingers, but no calcifications in the soft tissues.

The diagnosis of diffuse cutaneous systemic sclerosis (dcSSc) based on the revised criteria of LeRoy and Medsger [2], associated to classic adult PRP was made. The modified Rodnan skin thickness score was 26. A diligent search for underlying malignant disease was negative, and screening tests for hepatitis B, C and HIV were negative. Oral therapy with colchicine (1 mg per day) was instituted with partial improvement of skin manifestations. Il s’agit d’un gar殮 de 15 ans sans antécédents pathologiques particuliers qui a présenté une semaine avant son admission des douleurs abdominales diffuses avec vomissements alimentaires évoluant dans un contexte fébrile sans troubles de transit, ce qui a amené l’enfant à consulter et à être mis sous antibiotiques, sans amélioration.

L’examen clinique à l’admission a trouvé une température à 37°C avec sensibilité de la fosse iliaque gauche sans masse palpable, devant cette symptomatologie, un bilan biologique a été réalisé avec notamment une Numération Formule Sanguine (NFS) qui avait montré un taux d’hémoglobine à 11,8 g/dl, une hyperleucocytose à 17040 /mm3 à prédominance neutrophile, la C-reactive protein (CRP) était augmenté à 200 mg/l.

**Figure 3:**
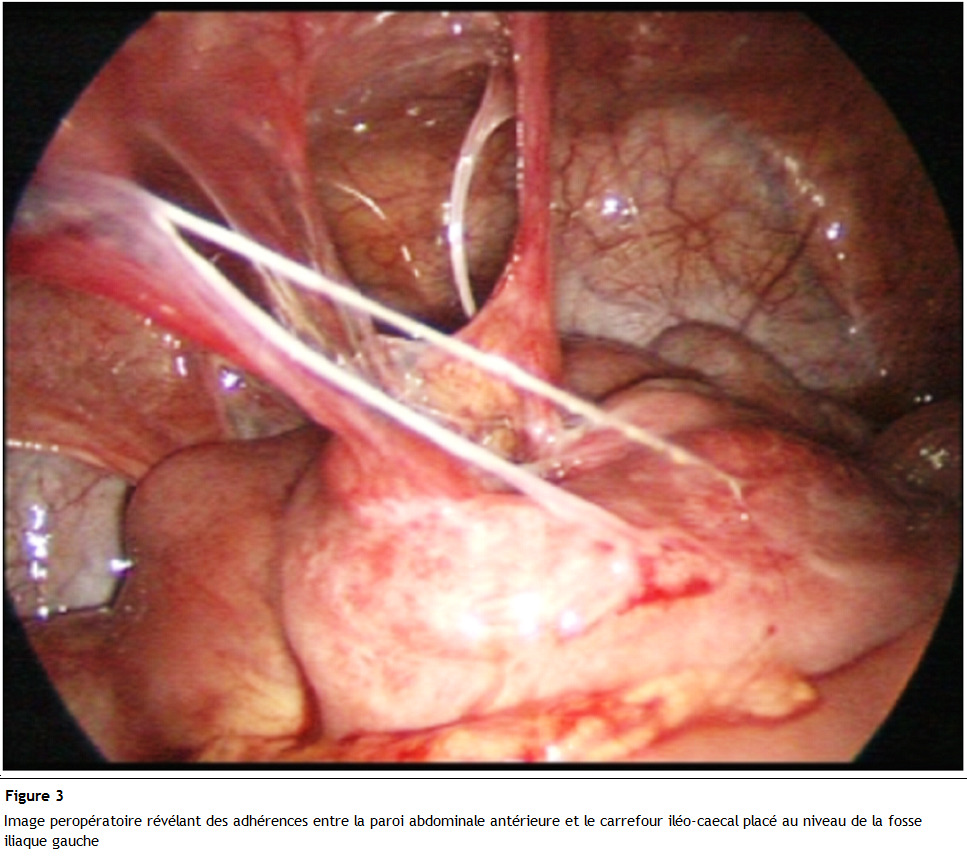
Coupe scannographique objectivant une transposition des vaisseaux mésentériques supérieurs

L’échographie abdominale a révélé une infiltration de la graisse avec multiples adénopathies et épanchement finement échogène de la fosse iliaque gauche; un lavement baryté a objectivé une projection à gauche du colon ascendant, du transverse et du colon descendant réalisant un aspect fortement évocateur d’un mésentère commun complet (Figure 1). Un complément scannographique a décelé une transposition des vaisseaux mésentériques supérieurs avec importante infiltration de la graisse mésentérique (Figure 2). Sur l’ensemble de ses arguments, il a été décidé une exploration cœlioscopique par un optique de 10 mm introduit au niveau de l’ombilic; ceci a mis en évidence quelques adhérences entre la paroi abdominale antérieure et le carrefour iléo-caecal placé au niveau de la fosse iliaque gauche (Figure 3) ainsi que de multiples adénopathies mésentériques. Un épanchement trouble de faible abondance a été prélevé pour étude cytobactériologique.

Le diagnostic de mésent	re commun complet a été confirmé du moment que tout le grêle siégeait à droite et le colon et l’appendice à gauche; ce dernier était très inflammatoire, boudinée. Nous avons procédé à l’aide de deux trocarts opérateurs de 5 mm, à l’aspiration du liquide de la fosse iliaque gauche puis à une appendicectomie après coagulation section du méso appendiculaire. Un lavage localisé au sérum salé 9 ‰ et enfin un drainage par un drain de redon ont été effectué.

Les suites opératoires ont été simples; l’étude anatomo-pathologique de la pièce opératoire est revenue en faveur d’une appendicite inflammatoire; l’évolution était favorable avec un recul de 6 mois.

## Discussion

Les anomalies congénitales du tractus gastro-intestinal sont une cause importante de morbidité chez les enfants et, moins fréquemment, chez les adultes [1]. Ces anomalies congénitales incluent les atrésies de l'intestin grêle et du côlon, les anomalies de rotation et de fixation, les anomalies ano-rectales et les duplications intestinales. Après une rotation normalement déroulé dans la vie intra-utérine, le mésentère a une base large qui s'étend de l'hypocondre gauche à la fosse iliaque droite.

La malrotation intestinale est une rotation anormale ou incomplète de l'intestin moyen responsable d’un mésentère raccourcie avec ligament de Treitz et caecum mal fixés [1]. Cette malrotation peut se compliquer d’un volvulus du grêle surtout en cas d’arrêt de la rotation à 180° [2] ou être asymptomatique lorsque l’arrêt se fait à 90° responsable donc d’un mésentère commun complet qui se complique rarement et dont la découverte est généralement fortuite lors des examens radiologiques réalisés pour d’autres indications.

L’observation qu’on rapporte révèle un autre mode de découverte de cette anomalie; une appendicite avec manifestation clinique gauche. L'appendicite est la cause d'environ un tiers des abdomens aiguës mais sa localisation gauche est extrêmement rare [3]. Une revue de la littérature récente rapporte 63 cas qui s’inscrivent dans le cadre d’un situs inversus [4-6]. Les cas rapportés dans le cadre d’une malrotation avec mésentère commun complet sont encore plus rares [7,8]; cette localisation inhabituelle est responsable d’un retard de diagnostic pouvant être à l’origine de complications graves. Avec l’avènement de l’imagerie moderne notamment l’échodoppler et la tomodensitométrie qui cherchent une transposition des vaisseaux mésentériques et l’emplacement du grêle par rapport au colon, le diagnostic se fait de plus en plus précocement [9-11].

La laparoscopie est primordiale dans ces cas puisqu’elle permet de confirmer l’anomalie anatomique puis proposer un traitement qui consisterait à une appendicectomie sous cœlioscopie [12]. En l’absence de cette technique, la tomodensitométrie (TDM) abdominale permet d’orienter la voie d’abord chirurgicale [9,13].

## Conclusion

Si l’appendicite est la pathologie chirurgicale abdominale la plus fréquemment rencontrée aux urgences pédiatriques, sa présentation atypique chez les patients ayant une malrotation intestinale présente un défi diagnostique. Il nécessite une imagerie appropriée reposant très souvent sur le scanner ainsi qu’une bonne connaissance des variabilités anatomiques du tube digestif. Le traitement a beaucoup bénéficié de la chirurgie laparoscopique.

## Competing interests

Les auteurs ne déclarent aucuns conflits d’intérêts.

## Authors’ contributions

Aziz Elmadia participé à la prise en charge du malade et à la rédaction de l’article, Hanan Bouamama a participé à la prise en charge du malade, Mohammed Rami et Khalid Khattala ont participé à la recherche bibliographique et à la prise en charge du malade, Abderrahman Afifi a participé à la prise en charge du malade, Youssef Bouabdallah a participé à la prise en charge du malade et à la rédaction de l’article.
